# Role of biomechanics in the understanding of normal, injured, and healing ligaments and tendons

**DOI:** 10.1186/1758-2555-1-9

**Published:** 2009-05-20

**Authors:** Ho-Joong Jung, Matthew B Fisher, Savio L-Y Woo

**Affiliations:** 1 Musculoskeletal Research Center, Department of Bioengineering, Swanson School of Engineering, University of Pittsburgh, Pittsburgh, USA; 2 Department of Orthopaedic Surgery, College of Medicine, Chung-Ang University, Seoul, Korea

## Abstract

Ligaments and tendons are soft connective tissues which serve essential roles for biomechanical function of the musculoskeletal system by stabilizing and guiding the motion of diarthrodial joints. Nevertheless, these tissues are frequently injured due to repetition and overuse as well as quick cutting motions that involve acceleration and deceleration. These injuries often upset this balance between mobility and stability of the joint which causes damage to other soft tissues manifested as pain and other morbidity, such as osteoarthritis.

The healing of ligament and tendon injuries varies from tissue to tissue. Tendinopathies are ubiquitous and can take up to 12 months for the pain to subside before one could return to normal activity. A ruptured medial collateral ligament (MCL) can generally heal spontaneously; however, its remodeling process takes years and its biomechanical properties remain inferior when compared to the normal MCL. It is also known that a midsubstance anterior cruciate ligament (ACL) tear has limited healing capability, and reconstruction by soft tissue grafts has been regularly performed to regain knee function. However, long term follow-up studies have revealed that 20–25% of patients experience unsatisfactory results. Thus, a better understanding of the function of ligaments and tendons, together with knowledge on their healing potential, may help investigators to develop novel strategies to accelerate and improve the healing process of ligaments and tendons.

With thousands of new papers published in the last ten years that involve biomechanics of ligaments and tendons, there is an increasing appreciation of this subject area. Such attention has positively impacted clinical practice. On the other hand, biomechanical data are complex in nature, and there is a danger of misinterpreting them. Thus, in these review, we will provide the readers with a brief overview of ligaments and tendons and refer them to appropriate methodologies used to obtain their biomechanical properties. Specifically, we hope the reader will pay attention to how the properties of these tissues can be altered due to various experimental and biologic factors. Following this background material, we will present how biomechanics can be applied to gain an understanding of the mechanisms as well as clinical management of various ligament and tendon ailments. To conclude, new technology, including imaging and robotics as well as functional tissue engineering, that could form novel treatment strategies to enhance healing of ligament and tendon are presented.

## Introduction

Ligaments and tendons are soft connective tissues which transmit forces from bone to bone and muscle to bone, respectively. These unique tissues serve essential roles for biomechanical function of the musculoskeletal system by stabilizing and guiding the motion of diarthrodial joints. To accomplish this, ligaments and tendons have a hierarchy of highly aligned collagen composed of fibrils, fascicles, fibers, and the tissue itself, to form one of the strongest tissues in the body. Nevertheless, these tissues are frequently injured due to repetition and overuse, eccentric activities, and quick cutting motions that involve acceleration and deceleration. These injuries often upset this balance between mobility and stability of the joint which results in abnormal loading that could damage other soft tissues in and around the joint manifested as pain and other morbidity, such as osteoarthritis [1-6].

The numbers on incidence of ligament and tendon injuries are huge. For example, it is estimated that tendinopathy accounts for 30% to 50% of all injuries related to sports, plus over 48% in occupational maladies [6-8]. Achilles tendinopathy accounts for 7–11% of all running injuries [9-11]. Similarly, the incidence of knee ligament ruptures, primarily involving the anterior cruciate ligament (ACL) and the medial collateral ligament (MCL), is estimated to be 2 per 1,000 people per year in the general population [12,13]. In the shoulder, injuries to the ligaments and capsule results in approximately 34,000 dislocations occur per year in the young adult population [14,15], and rotator cuff tears in more than 30% of people over 60 years of age [16,17].

The healing of ligament and tendon injuries, if it takes place, is usually slow. Tendinopathies, including tendinosis, tendonitis, and paratenonitis, are ubiquitous, occurring in the Achilles, patellar, quadriceps, hamstrings, and rotator cuff tendons as well as in the elbow, wrist and hand joints. These detrimental injuries can take up to 12 months for the pain to subside before one could return to physical and sports activity [6]. Further, although a ruptured MCL can generally heal spontaneously and sufficiently well such that nonsurgical management has become the treatment of choice [18-25], its remodeling process takes years, and its mechanical properties remain inferior to those for the normal MCL [18,23,24,26-30]. It is also known that a midsubstance ACL tear has limited healing capability [20,31-33] and reconstruction by replacement grafts has been regularly performed in order to regain knee function [34-37].

Well over 100,000 of these procedures are done per year in the U.S. alone [13]. However, long term follow-up studies have revealed that 20–25% of patients experience unsatisfactory results at 7 to 10 years following ACL reconstruction [1-5]. Thus, it is clear that a better understanding of the function of ligaments and tendons, together with knowledge on their biology and healing potential, is needed for investigators to develop novel strategies to accelerate and improve the process of healing of ligaments and tendons. Well over 4000 new papers have been published in the last ten years that involve biomechanics of ligaments and tendons. As a result, orthopaedic surgeons and other health related professionals have an increasing appreciation of the importance of this subject area and have use for biomechanical findings to help their clinical practice. On the other hand, biomechanical data are complex in nature, and there is a danger of misinterpreting them. Thus, we wish to provide the readers with a brief overview of ligaments and tendons and point them to appropriate methodologies used to obtain the biomechanical properties of these soft tissues [38-43]. More importantly, how the properties of these tissues can be altered due to various experimental and biologic factors will be discussed [44-52]. Following this background material, we will present how biomechanics can be applied to gain an understanding of the mechanisms and management of various ligament and tendon injuries. Examples given will include tendinopathy, as well as MCL and ACL tears. To conclude, new technology, including imaging and robotics as well as functional tissue engineering, that could form novel treatment strategies to enhance ligament and tendon healing will be presented [53-61].

### Basic biomechanics on ligaments and tendons

#### Definition of biomechanical properties

**Figure 1:**
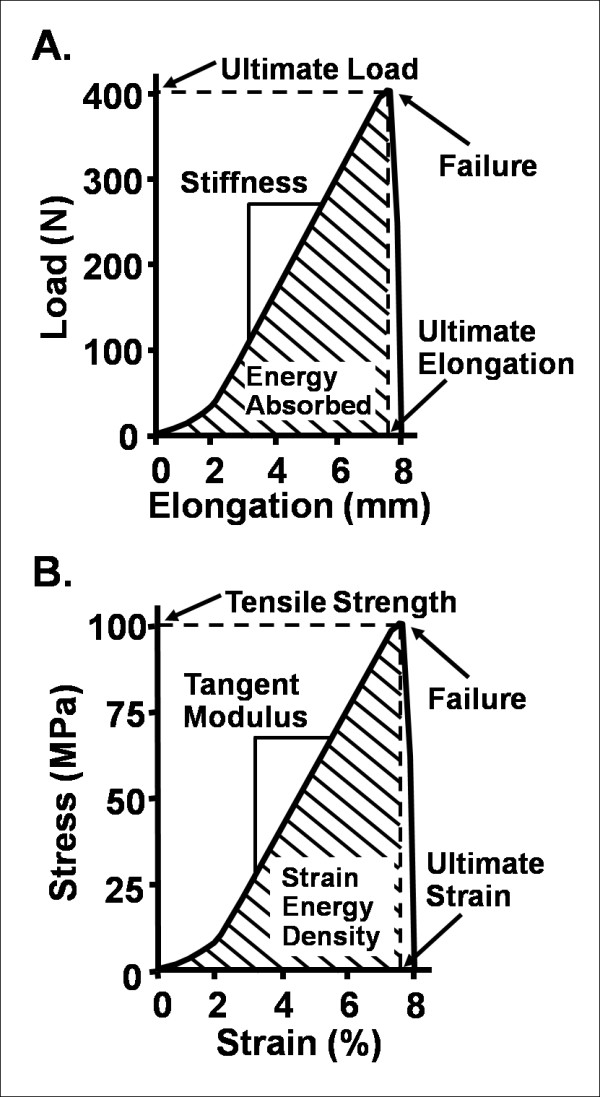
**A: A representative load-elongation curve of the bone-ligament-bone or muscle-tendon-bone complex**. **B: **A stress-strain curve representing the mechanical properties of a ligament or tendon substance.

Because the primary function of ligaments and tendons is to transmit tensile forces, experimental studies of the biomechanical properties of these tissues are generally performed in uniaxial tension. Testing isolated ligament and tendon tissue is inherently difficult for several reasons (i.e., slipping of the specimen from the clamp, stress concentrations, shortness of substance of ligaments). As a result, tensile tests have been performed with the ligament or tendon insertions to bone left anatomically intact (e.g., the entire bone-ligament-bone complex). From this test, a nonlinear (concave upwards) load-elongation curve for the bone-ligament-bone complex or muscle-tendon-bone complex can be obtained (Figure 1A). Parameters obtained from this curve, representing the structural properties of the complex, include stiffness, ultimate load, ultimate elongation, and energy absorbed at failure.

From the same uniaxial tensile test, a stress-strain curve of the ligament or tendon substance (from which its mechanical properties are determined) can also be obtained. This is done by normalizing the tensile load by the cross-sectional area (i.e., stress) and by normalizing the change in elongation in a defined region of the tissue midsubstance by the initial length (i.e., strain) (Figure 1B). One assumption in this test is that of a uniform stress distribution throughout the specimen. By engineering standards, this requires an aspect ratio (ratio of length to width) of greater than 10 to 1. However, for biological tissues, a ratio of greater than 4 to 1 is generally accepted due to anatomical limitations. Parameters obtained from this curve, representing the mechanical properties, or quality, of the tissue substance include the tangent modulus, ultimate tensile stress (or tensile strength), ultimate strain, and strain energy density of the ligament or tendon substance.

#### Methods to determine stress and strain

As a specimen's cross-sectional area is required for stress calculations, methods to accurately measure it are important. However, the irregular, complex geometry presents challenges, and in some cases, can introduce very large errors. The literature has presented us with a long list of techniques [62-65]. In general, they can be divided into contact or noncontact approaches [42,66]. Contact methods include molding techniques, digital vernier calipers, and area micrometers [67]. We, as well as many others, have advocated the use of noncontact methods, so as not to disturb the natural shape of these soft tissues. These techniques include the shadow amplitude method [62], the profile method [64], and the use of a light source [63]. In our research center, laser micrometer systems have been developed to measure both the cross-sectional area and the shape of soft tissues with high accuracy and precision [42]. For those tissues that contain concavities, we have developed a more advanced laser reflectance system to accurately measure cross-sectional shape and area of these soft tissues [66].

Accurate experimental measurement of tissue strains also poses a number of hurdles. Methods, such as video tracking systems, have been developed to avoid possible errors of elongation from contribution of connecting structures [68-71]. Two or more reference markers are placed on the surface of the tendon or ligament substance, by means of Verhoeff's stain, elastin stain, or reflective tape, to serve as gauge lengths [23,50]. Then, a camera system could record the motion of the markers during tensile stretch, and the percentage strain of the tissue could be calculated [70]. Additionally, it must be noted that under tension, the strain along the ligaments and tendons can vary considerably. We have found that higher strains occur near the insertion sites [72]. To measure tissue elongation in-vivo, contact techniques such as the use of a differential variable reluctance transducer have been employed to obtain in-vivo strain of the ACL during physiologic motions [73].

The range of mechanical properties of various ligaments and tendons is very large as these tissues are designed specifically to function in different joints (Table 1) [67,74-85]. For the human MCL, the tangent modulus and the tensile strength were found to be 332.2 ± 58.3 MPa and 38.6 ± 4.8 MPa, respectively [74]. In comparison, values for the inferior glenohumeral ligament were found to be many folds lower than those for the MCL, ranging from 5 to 42 MPa and 1 to 6 MPa, respectively [83]. The large range of motion and flexibility of the glenohumeral joint dictate such lower values.

Additionally, since ligaments and tendons are highly organized fibrous tissues, their mechanical properties are directionally dependent (anisotropic). For example, the human MCL has mechanical properties that are 30 times higher along its longitudinal direction compared to its transverse direction [74].

**Table 1:** Mechanical properties of human ligaments and tendons

Tissue	Modulus (MPa)	Ultimate Tensile Strength (MPa)	Ultimate Strain (%)	Source
Knee				
Medial Collateral Ligament	332 ± 58	39 ± 5	17 ± 2	[74]
Anterior Cruciate Ligament	65–447	13–46	15–44	[75-77]
Posterior Cruciate Ligament	150–447	30–36	11–19	[67,75,78]
Patellar Tendon	504–680	54–65	12–15	[75,79,80]
Lower extremity				
Gracilis Tendon	590–1734	111–112	7–19.4	[79,81]
Semitendinosus Tendon	540–1081	89–124	8–23	[79,81]
Achilles Tendon	819 ± 208	79 ± 22	9 ± 2	[82]
Shoulder				
Inferior Glenohumeral Ligament	5–42	1–6	8–33	[83,84]
Posterior Glenohumeral Capsule	10–32	2–5	22–23	[85]

#### Determination of viscoelastic properties

Ligaments and tendons display time- and history-dependent viscoelastic properties that reflect the complex interactions between proteins (e.g. type I, III, V collagen, elastin), ground substance (e.g. proteoglycans and glycolipids), and water. The loading and unloading curves of these tissues do not follow the same path but instead form a hysteresis loop representing internal energy dissipation during each cycle. Additional important viscoelastic characteristics of ligaments and tendons are creep (i.e., an increase in deformation over time under a constant load) and stress relaxation (i.e., a decline in stress over time under a constant deformation). These viscoelastic behaviors for ligaments and tendons have important clinical significance as they help to prevent fatigue failure of ligaments and tendons. For example, during walking or jogging, cyclic stress relaxation occurs, in which the peak stress in the tissue substance decreases with each cycle [7,86].

Mathematical models have been made to describe the viscoelastic properties of ligaments and tendons. The quasi-linear viscoelastic (QLV) theory originally formulated by Fung (1972) has been modified and adopted to describe the viscoelastic properties of the normal and healing MCL and ACL [87-96]. For ligaments and tendons undergoing larger deformations, a single integral finite strain (SIFS) theory has been implemented and used for the patellar tendon and ACL [97]. The interested readers are referred to others research articles and reviews for a more in-depth treatment of this subject area [88-90].

#### Factors influencing tensile properties of ligaments and tendons

When tensile testing ligaments and tendons many factors can impact the outcome of their biomechanical properties. Those factors can be divided into 1) experimental factors, and 2) biological factors and can help the readers to identify why the findings on the same tissue can differ from study to study.

Specimen orientation during tensile testing, e.g. for the human femur-ACL-tibia complex (FATC), can alter its structural properties significantly because of its geometric complexity [48,98]. When tested in an anatomic orientation, by maintaining its natural insertion angles, the results became significantly differ from those tested in an orientation along the tibia [48]. For example, the ultimate load of specimens in the anatomic orientation was 35% higher than those in the tibial orientation in young donors.

Considerable attention has also been given to the effects of the strain rate on the mechanical properties of ligaments and tendons [98-100]. Using skeletally mature rabbits, we could demonstrate little rate sensitivity over a range of four and a half decades of strain rates [98,101]. Other environmental conditions, including temperature and hydration, however, were found to have significant influence on the mechanical properties of ligaments and tendons [49,102]. Finally, appropriate storage by freezing with the muscles and joint intact were found to have little or no significant differences on its biomechanical properties after one or two freeze-thaw cycles. It should be cautioned that special care still must be taken in tissue sample storage, especially relating to protecting tissue before and after freezing from a lack of hydration [50,103,104].

Age-related changes are one biological factor worth discussing. Skeletal maturity has been shown to play a major role in the biomechanical properties of tendons and ligaments, because of the development at their insertions. For New Zealand white rabbits, the structural properties of the femur-MCL-tibia complex (FMTC) increased dramatically with skeletal maturity. For example, the stiffness in the skeletally mature group (12–15 months of age) were 40% higher than skeletally immature animals (4–6 months of age). Similar results were obtained for the mechanical properties of the ligament substance, as up to 90% increases in modulus were observed with skeletal maturity [51,105].

**Figure 2:**
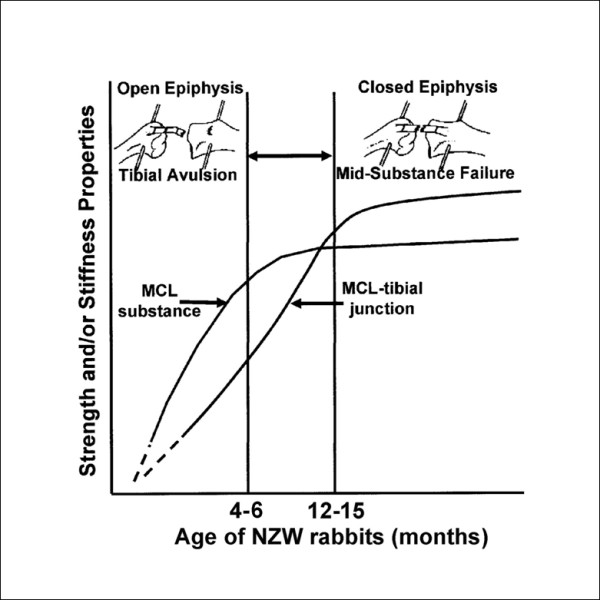
**A schematic diagram depicting the relationship between failure mode and age, hypothesizing the asynchronous rates of maturation between the bone-ligament-bone complex and the ligament substance (permission requested from **[51]).

All skeletally immature FMTCs failed by tibial avulsion; whereas, the majority of skeletally mature animals failed at the tissue substance indicating that there is an asynchronous maturation process between the bone-ligament-bone complex and ligament midsubstance [51] (Figure 2). Prior to skeletal maturity, the strength of the MCL substance reached near its peak value, while the bone-ligament junction, especially at the proximal tibial insertion site, was still maturing, causing failure by tibial avulsion. Once skeletal maturity was reached, the proximal tibial insertion site became stronger, and the FMTC failed in the ligament substance.

The properties of ligaments and tendons also can change with advancing age as well as with activity level [83,106,107]. For example, in the human FATC, the mean stiffness and ultimate load of younger specimens (22–41 years) was 1.3 and 3.3 times higher, respectively, than those obtained from older specimens (60–97 years) [48].

**Figure 3:**
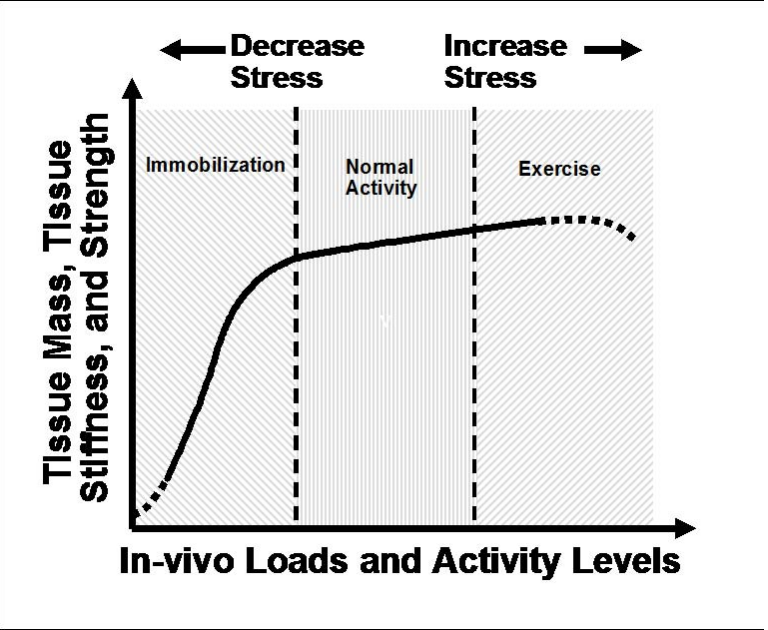
**A schematic diagram describing the homeostatic responses of ligaments and tendons in response to different levels of stress and motion (permission requested from **[47]).

Ligaments and tendons are also known to remodel according to applied motion and stress. Studies using rabbit hind limbs have shown that a few weeks of immobilization can severely increase joint stiffness as well as significantly decrease the structural properties of the FMTC and the mechanical properties of the MCL tissue [47]. Remobilization could reverse these negative changes, but would require up to one year before these biomechanical properties could return to near normal levels [47]. On the other hand, exercise and training could only result in marginal increases in the biomechanical properties of ligaments (e.g. MCL of the knee) and tendons (e.g. extensor and flexor tendons of the hand) [45,52,109]. Based on these studies, a highly nonlinear curve could be drawn to represent the relationship between levels of stress and motion with the changes in properties for ligaments and tendons (Figure 3). Following immobilization, there would be a rapid reduction in tissue properties and mass from those for the normal range of physiological activities- as represented by the middle of the curve. In contrast, the positive gains following long-term exercise and training are much more moderate.

### Biomechanics and tendinopathy

Tendinopathy, including tendinosis, tendonitis, and paratenonitis, is a work related and/or sports-induced ailment that involves severe discomfort and pain [9,110-115]. In general, tendons, after being subjected to repetitive loading of varying levels and for a prolonged period of time, could exhibit chronic pathology. Nevertheless, the etiology of tendinopathy is still unclear and hotly debated. Many years of research suggest that the problem is likely multifactorial [6,116-118]. Biomechanically, it is thought that microtrauma first occurs in the tendon, causing rupture of a small number of collagen fibrils while increasing the loading of the remainder [117-121]. The initial insult could be either due to one abnormal loading cycle of high (but subfailure) strain or a series of lower subfailure strains [70,110,122-129]. A number of in-vitro studies have shown that both loading regimens can induce such injury and the ensuing degeneration of tendons, is characterized by a reduction in their biomechanical properties [130-132].

A mechanism by which the trauma spreads to the remaining intact tendon until the symptoms of tendinopathy are presented has been suggested by a number of in-vitro experiments [110,116,119,128,133-136]. These studies have shown that high strains (or long duty cycles) applied by stretching bioreactors can cause tenocytes to produce abnormal levels of matrix metalloproteases (MMPs), which would in turn degrade the collagen and cause degeneration [131,137-144]. However, other studies have shown that loading protocols consisting of large strains were well-tolerated by tendon fibroblasts [6]. Whether these in-vitro strain levels simulate the physiological condition, and whether tensile loads that are within or slightly above the normal physiological range would cause any damage to the tendon fibroblasts are hotly debated [117]. Further, imaging studies of common sites of tendinopathy, e.g. for the supraspinatus tendon, patellar tendon, and Achilles tendon, were found to be in relatively lower levels of tensile strains than other parts of the tendon, suggesting that high tensile strains may not be the only major etiologic factor [116,145,146].

Recently, in-vitro studies have demonstrated that understimulation of tenocytes can also lead to altered MMP expression and apoptosis, followed by tendon degeneration [147-152]. Others hypothesized that it is the compressive loading that leads to altered cellular morphology and degenerative changes [116,120,153-156]. Additionally, internal shear forces and heat have been postulated to be causes of intratendinous degeneration [6]. New finite element models of tendons may be useful tools to predict the response of these various loads on tendon cells and the resulting changes in extracellular matrix [157]. The literature has also shown other etiological factors, such as age, and gender, in the development of tendinopathy. Interested readers are referred to the International Olympic Committee Medal Commission publication *Tendinopathy in Athletes *edited by Woo, Renström, and Arnoczky for additional details [6]. Overall, it is clear that tendinopathies are complex biomechanical and biochemical problems.

#### Management of tendinopathy

Clinical management of tendinopathies are frequently empirically-based, and as such, the recommended treatment strategies by different clinicians can vary greatly. Although excessive exercise may be a cause of tendinopathy, eccentric exercise has been shown to work well in a number of studies to treat tendinopathy [112,158,159]. More than 80% of patients were satisfied with the treatment and were able to return to previous activity levels [159-161]. Others have used traditional Chinese exercises, such as Tai Chi, to enhance body functions in promoting muscle performance, increasing flexibility and proprioception while weight bearing to stimulate bone and soft tissue [6]. These treatment strategies are supported by previous animal studies which have shown that moderate, prolonged exercise is effective and results in an increase in the cross-sectional area of swine extensor tendons as well as its mechanical properties, indicating improved tissue quality [109], as shown in Figure 3[162,163]. These changes contribute to the training-induced adaptation in mechanical properties, whereby exercise could improve tolerance toward strenuous exercise to avoid injury.

Additional modalities such as ultrasound, laser photostimulation, deep heat, pulsed magnetic and electromagnetic fields, and electrical stimulation are also used to treat tendinopathy [164-167]. Application of these modalities is intended to affect the stiffness of newly formed scar tissue inside the tendon. Yet other investigators use intratendinous injections of corticosteroids to relieve pain, but these injections may lead to negative effects on the mechanical properties [168-171].

When conservative treatment has failed, surgeons have used many techniques including needling, coblation, percutaneous tenotomy, arthroscopic debridement, percutaneous paratenon stripping, open tenotomy, paratenon stripping, and tendon grafting to treat tendinopathy [172]. In general, a return to sports after surgery has been reported in 60–85% of cases, although some have questioned these findings [173]. It has been suggested that first time ailments may require 2–3 months to recover but chronic cases may take 4–12 months [174]. As the physiologic, biomechanical, and biologic mechanisms of this ailment are not clearly understood, the general clinical advice remains that one must be patient as there is no "quick fix."

### Biomechanics and ACL reconstruction

For the ACL, it is well known that there is limited capacity for healing after its injury [36,175-177]. Unlike extraarticular ligaments, there exist several well known factors that limit the ACL from healing [178,179]. The thin synovium surrounding the ACL, which has been shown to play an important role in providing a vascular supply to the relatively avascular ACL as well as to protect it from the harsh synovial fluid [180], is disrupted and not regenerated until 1 to 2 months following ACL injury [175,181-183]. Histological examination of the human ACL reveals that it is also retracted following rupture and that clots formed are insufficient to fill the open gap [175]. In addition, a number of studies have also documented that the intrinsic healing capacities, cellular proliferation, and extracellular matrix (ECM) production of the ACL are also lower compared to the MCL [24,178,179,181,184-188].

Most studies show unfavorable results after conservative treatment of an ACL rupture, particularly in patients with high levels of activity [189-193]. Overall success rate of conservative treatment for ACL has been about 30 to 50% [37,189,194,195]. Surgical replacements have been done for a large percentage of patients to help maintain knee stability [196-200]. Autografts, such as the bone-patellar tendon-bone (BPTB) and hamstring tendons, and allografts are the graft of choice. However, long-term follow-up studies (10+ years) of patients with ACL reconstruction showed 20–25% unsatisfactory results, with complaints such as knee pain and extension deficits [78,201-204], and most concerning, many of these cases had progressed to knee osteoarthritis [205-217]. Much effort is being made to improve the clinical outcome. Biomechanical studies can be used to lead to better understanding of the kinematics of the knee and the function of the intact ACL and ACL replacement grafts in order to improve surgical decision making.

#### Complex anatomy of the ACL and its role in knee function

In the human knee, the ACL can be represented by two bundles: the anteromedial (AM) and the posterolateral (PL) bundle. The anatomic division of these bundles is based on the gross tensioning pattern of the ACL during passive flexion-extension of the knee, with the AM bundle tauter in flexion and the PL bundle tauter in extension. With these two bundles, the ACL is well designed to stabilize the knee throughout knee flexion under various loading conditions.

**Figure 4:**
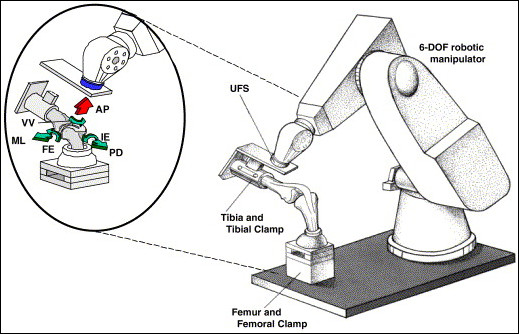
**Schematic drawing illustrating a robotic/universal force-moment testing system and the six degrees of freedom of motion of the human knee joint (permission requested from **[218]).

To assess the function of the ACL under multiple degree of freedom (DOF) knee motion, our research center has developed a robotic/universal force moment sensor (UFS) testing system since 1993 (Figure 4) [218]. This novel testing system can control and reproduce the multiple DOF knee motion [38,40,41,219-221] and is also capable of applying external loads to knees. By operating in both force and position control modes, the robot can apply a pre-determined external load to the specimen, such as those used for the diagnosis of ACL deficiency [222-224], and the corresponding kinematics can be obtained. For example, an 134-N anterior tibial load can be applied to simulate the Lachman and anterior drawer tests, or a combined 10 N·m of valgus torque and 5 N·m of internal-external tibial torque can be applied to statically simulate the pivot-shift test. Then, the ACL can be transected. Subsequently, the specimen is moved along a previously recorded path of motion while the UFS records a new set of force and moment data [38]. Since the path of motion can be precisely repeated, the in-situ force in the ligament can be calculated by determining the changes in forces after cutting the ACL, based on the principle of superposition [41].

Most importantly, this advanced methodology has the advantage of collecting experimental data from the same cadaveric knee specimen under different experimental conditions (such as ACL intact, and ACL-reconstructed knee conditions), eliminating the effect of interspecimen variation. Thus, the robotic/UFS testing system has been used to quantitatively determine the data on motions of the intact, ligament deficient, and reconstructed knee with respect to the same reference position while the in-situ forces in ligaments and replacement grafts were calculated. As a result, the analysis of data through repeated-measures analysis of variance significantly increases the statistical power. To date, over 80 studies have been published using this technology, and it has been adopted by many laboratories around the world [225,226].

We have found that the two anatomical bundles of the ACL have different functions even under the simplest external loading conditions [220]. For instance, it has been found that under an anterior tibial load, the PL bundle carried a higher load than the AM bundle with the knee near extension, and the AM bundle carried a higher load with the knee flexion angle larger than 30 degrees. It was also found that when the knee was subjected to combined rotatory loads of valgus and internal tibial torques to statically simulate the pivot shift test, the AM and PL bundles almost evenly shared the load at 15 degrees of knee flexion. Thus, the fact that both bundles play significant roles in controlling knee stability is well illustrated.

**Figure 5:**
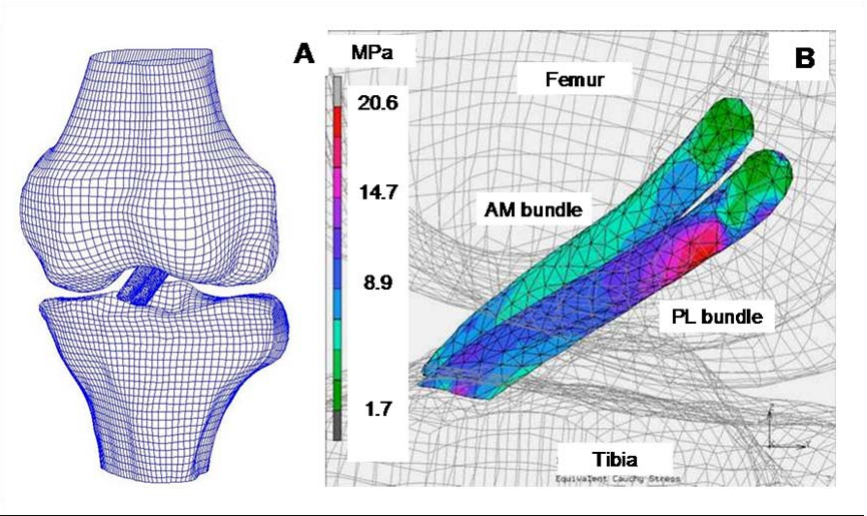
**A) Finite element model of the knee joint and B) Cauchy stress distribution within the AM and PL bundles under a 134 N anterior tibial load with the knee at full extension (lateral view). (permission requested from **[228]).

Computational finite element models are also valuable for studying for the complex function of the ACL and its bundles. Once such models are validated with experimental data (e.g., knee kinematics or in-situ forces as determined using the robotic/UFS testing system [227,228]), they can be used to calculate the stress and strain distribution in the ACL, by incorporating its non-uniform geometry (in which the midsubstance cross-sectional area is one-third that of the insertions) and its twisting fiber orientation. For example, it could be shown that the peak stresses in the ACL were seen near the femoral insertion sites, suggesting this area could be susceptible to injury (Figure 5). These results are consistent with clinical reports in which most ACL tears occur near the femoral insertion site [229].

#### Choice of autograft

As the majority of autografts for ACL reconstruction procedures are either bone-patellar tendon-bone or hamstrings tendon autografts, it is of interest to compare their biomechanical effectiveness to restore knee stability. To accomplish this goal, human cadaveric knees were tested using the robotic/UFS testing system following ACL reconstruction using a hamstrings tendon graft or bone-patellar tendon-bone graft [54]. Due to this unique system, both reconstructions were performed in the same knees and could be quantitatively compared. When an anterior tibial load was applied, both reconstructions were found to be successful in limiting excessive anterior tibial translation, and the in-situ forces in the grafts were near those of the intact ACL from full extension to 90° of flexion. However, when a combined 10 N-m valgus and 5 N-m internal tibial torque was applied, both reconstructions were insufficient to reduce coupled anterior tibial translation. This insufficiency was further revealed by the significantly lower in-situ forces in the grafts, which ranged from 45% to 65% of that in the intact ACL. Thus, our data suggest better graft placement, i.e. the location of the femoral tunnel such that the graft could resist rotatory loads applied to knee, would be needed.

#### Femoral tunnel location

To investigate the effect of femoral tunnel location for ACL reconstruction grafts, a series of biomechanically based experiments have been performed in our research center [54,55,230]. First, we studied how well a bone-patellar tendon-bone autograft placed at the PL or AM insertion site in the femur (equivalent to the 10 and 11 o'clock positions in the frontal view) could restore knee function [231]. In response to an anterior tibial load, both the single PL-bundle and single AM-bundle reconstructions were able to restore anterior tibial translation to a level similar to that of the intact knee, except at deep knee flexion angles for the single PL-bundle reconstruction. Under combined rotatory loads, however, the single PL-bundle reconstruction could better restore the coupled anterior tibial translation and in-situ force in the graft to those of the intact knee at 15° and 30° of knee flexion. Thus, although the grafts are similarly effective under an anterior tibial load, the more laterally placed single PL-bundle reconstruction could more effectively resist rotatory loads, particularly when the knee is near full extension.

We then considered a double bundle reconstruction by replacing both the AM and PL bundles so that it would better approximate the anatomy of the ACL, and compared it to a single AM bundle reconstruction. The double bundle reconstruction could not only better restore knee kinematics and in-situ force under anterior tibial loading but also under the combined rotatory loading. Additionally, the in-situ force at 30° of flexion was 91% ± 35% of the intact ACL, compared to only 66% ± 40% for the single AM bundle reconstruction under the rotatory loads.

**Figure 6:**
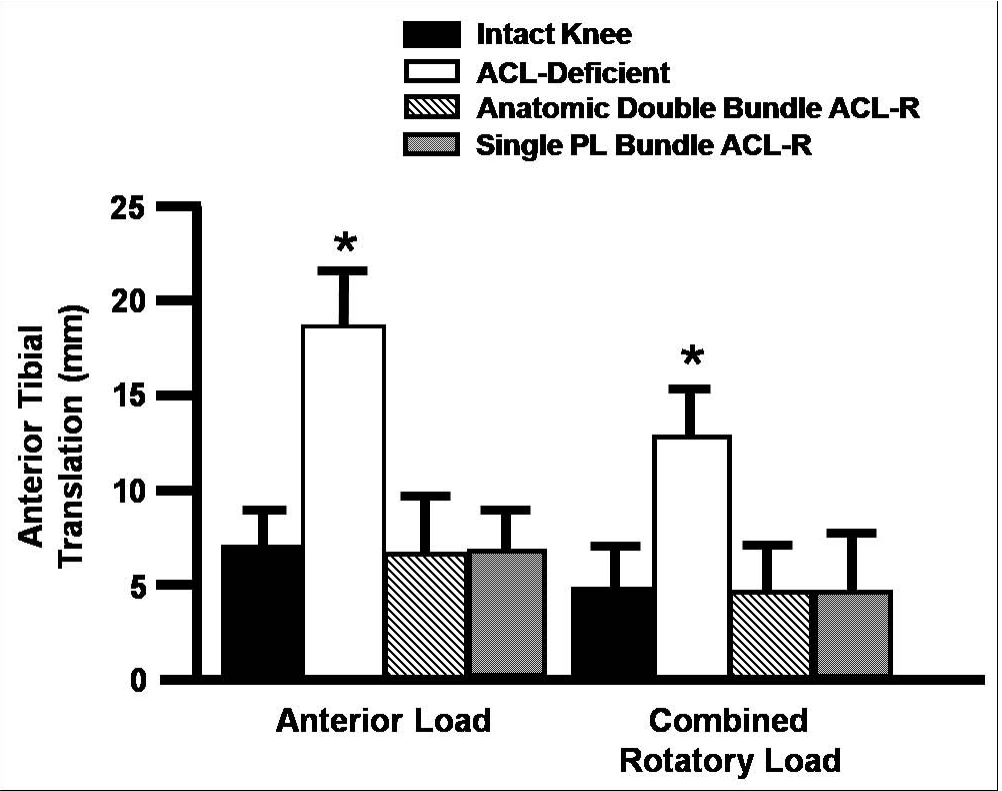
**Anterior tibial translation (mean ± SD) in the intact, anterior cruciate ligament (ACL)-deficient, and anatomic double bundle and single posterolateral (PL) bundle ACL-reconstructed (ACL-R) knees in response to an anterior tibial load and combined rotational load at 15° of knee flexion**. An asterisk indicates a statistically significant difference (p < 0.05) (permission requested from [232]).

A similar study was also done to compare the anatomic double bundle reconstruction to a more laterally placed single PL bundle reconstruction [232]. In this case, in response to anterior tibial and combined rotatory loads, both reconstructions were able to restore anterior tibial translation and in-situ force in the ACL graft near those for the intact knee at flexion angles less than 90° of knee flexion. Moreover, there were no significant differences between the two reconstruction procedures at 15° and 30° of knee flexion under either loading condition (Figure 6, p < 0.05). Thus, to reproduce the complex function of the ACL throughout the range of knee flexion, reproducing both bundles of the ACL may have some biomechanical advantages. On the other hand, a more laterally placed reconstruction, such as the single PL bundle reconstruction, may also work well, especially with the knee near extension where the function of the ACL is most critical.

### Biomechanics and ligament healing of the MCL

Laboratory research has discovered that the injured MCL of the knee can heal spontaneously [24]. Conservative treatment of an isolated MCL injury has produced better or similar results to those with surgical repair either with or without immobilization, in terms of restoring varus-valgus knee stability and its biomechanical properties [24,34]. Immobilization after ligament injury was shown to lead to a greater percentage of disorganized collagen fibrils, decreased structural properties of the FMTC, decreased mechanical properties of the ligament substance, and slower recovery of the resorbed insertion sites [47]. As a result, for the last twenty-five years the paradigm of clinical management of MCL tears has shifted from surgical repair with immobilization to functional management with early controlled motion [186,233].

The MCL has been an excellent experimental model to help understand the rate, quality, and composition of healing ligaments and tendons as well as treatment modalities [24,234]. It has brought better understanding of the continuous process of healing. Roughly, it can be divided into three overlapping phases [24,72]. The inflammatory phase is marked by hematoma formation which starts immediately after injury and lasts for a few weeks. It is followed by the reparative phase where fibroblasts proliferate and produce a matrix of proteoglycan and collagen, especially type III collagen, to bridge between the torn ends. Over the next 6 weeks, an increasingly organized ECM formation, predominantly type I collagen, and cellular proliferation occur. Finally, the remodeling phase, which is marked by alignment of collagen fibers and increased collagen matrix maturation, can continue for years [72].

**Figure 7:**
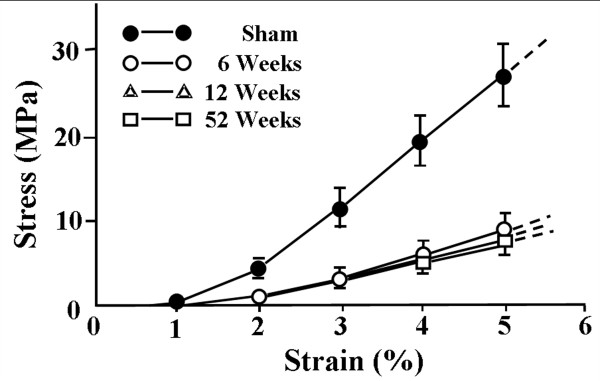
**Stress-strain curves representing the mechanical properties of the medial collateral ligament substance for sham-operated and healing MCLs at time periods of 6 (n = 6), 12 (n = 6), and 52 (n = 4) weeks (permission requested from **[22]).

These changes are reflected in the structural properties of the healing FMTC which are inferior to controls at 12 weeks after injury [24]. However, by 52 weeks post-injury the stiffness of the injured FMTC recovered, but the ultimate load remained lower than those for the sham-operated MCL [22,235,236]. This return to normal is largely due to an increase in the cross-sectional area of the healing ligament to as much as 2 1/2 times its normal size [237]. Thus, the recovery of the stiffness of the FMTC is largely the result of an increase in tissue quantity. On the other hand, the mechanical properties of the healing MCL midsubstance remain consistently inferior to those of the normal ligament and do not change with time up to one year [24,237] (Figure 7). This means the healing process involves a larger quantity of lesser quality ligamentous tissue.

Clinical management of combined ligamentous injuries to the knee is obviously more difficult and remains controversial [20,186,238,239]. In the case of a combined ACL/MCL injury, our research center has utilized basic science studies to elucidate suitable treatments. As a first step, it is important to understand the contribution of each ligament to knee stability. For example, under a valgus torque, there was a 123% increase in valgus rotation of the knee after the ACL was transected in contrast to only a 21% increase when the MCL was transected, when the knee was allowed to move freely in 5 DOF (varus-valgus and internal-external tibial rotation, proximal–distal, anteroposterior, and medial–lateral translations) [240]. The MCL was only found to be the "primary" restraint to valgus rotation when the knee joint was artificially restrained to 3 DOF, i.e. axial tibial rotation and anteroposterior translation. As a result, after a MCL injury, the functional deficit in valgus rotation can be compensated for by the remaining structures, especially the ACL.

In a combined ACL/MCL injury model in the rabbit, it was demonstrated that MCL repair combined with ACL reconstruction restored valgus laxity and improved the structural properties of the FMTC over those with ACL reconstruction alone in the short term (12 weeks). However, at longer term (52 weeks), the advantages of the MCL repair on the biomechanical properties of the FMTC disappeared [30,237]. Therefore, it is apparent that only reconstruction of the ACL is necessary after a combined injury due to the important role the ACL plays in maintaining valgus instability.

Further studies of combined ACL/MCL injuries in the goat model using our robotic/UFS testing system have shown that the initially high in-situ force in the ACL graft was transferred to the healing MCL during the early stages of healing (i.e. from time zero to 6 weeks). These excessively high loads likely contributed to the observed decrease in the structural properties of the FMTC and tangent modulus of the MCL substance when compared to an isolated MCL injury [23,241]. Thus, future work is still needed to optimize treatment strategies following combined ligamentous injuries.

### Summary and future directions

During the past four decades, significant advances have been made in characterizing the biomechanical properties of normal and injured ligaments and tendons. Data on the tensile and viscoelastic properties of ligaments and tendons, as well as knowledge on their contribution to joint kinematics and function, have significantly helped to move the field forward. Further, new findings on the healing process have led to improved treatment of ruptured ligaments and tendons, making it an exciting period for ligament and tendon research.

The new field of functional tissue engineering offers yet many new possibilities. The use of growth factors, gene therapy, cell therapy, and biological scaffolds to enhance healing certainly will result in improved outcomes [61,242-247]. We believe the ECM-derived bioscaffolds will play a significant role because it could accelerate the healing process and establish a bridge between the torn ends. ECM bioscaffolds have been successfully applied to improve MCL and PT healing, because their chemoattractant degradation products and bioactive agents allow aligned matrix formation with a concomitant improvement in biomechanical properties closer to normal values [58,248-250]. Efforts to healing of the ACL, which has long been considered as a "holy grail" for orthopaedics are now being made [251,252]. Additionally, it is possible to further improve the properties of these biological scaffolds by seeding bone marrow-derived cells (BMDCs) in-vitro followed by mechanical cyclic stretching to achieve better fiber alignment, thus making them to better suited to accelerate the healing process when used in-vivo [253].

**Figure 8:**
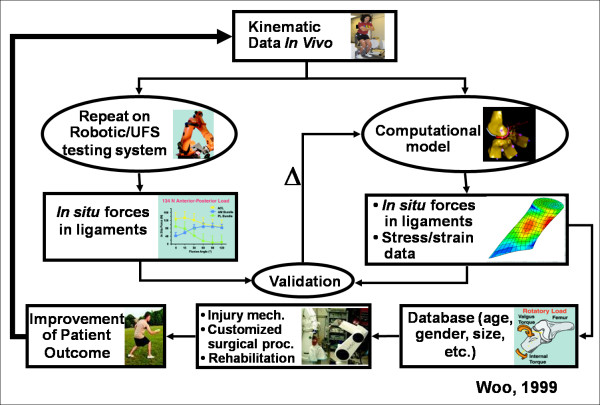
**Flow chart showing the utilization of in-vivo kinematics data to drive experimental and computational methodologies leading to improved patient outcome (permission requested from **[221]).

While in-vitro testing has contributed significant advances, it is time to move on to in-vivo studies. To do this, we have developed a new higher payload robotic/UFS testing system capable of handling levels of applied loads during activities of daily living (Figure 8). This system has also been shown to be highly accurate to 0.1 mm and 0.1°, which is needed to obtain meaningful data. Additionally, our collaborators at the Steadman-Hawkins Research Foundation have constructed a new biplanar fluoroscopy system, designed to measure in-vivo joint kinematics. It could measure the knee motion to within an accuracy of less than 0.2 mm and 0.3° [253]. These accurate in-vivo kinematics data can then be reproduced on cadaveric knees utilizing the robotic/UFS testing system and the in-situ forces in the ACL can be obtained. Thus, the combined technology will allow us to identify mechanisms of ACL (as well as other ligament and tendon) injuries and to design and optimize reconstruction procedures and rehabilitation protocols leading to improved patient outcomes as well as to help athletes successfully return to sports.

Moreover, in-vivo kinematics can be used as inputs for a three-dimensional finite element model of the knee. The predicted results from the model must be first validated with experimental data such as those obtained from the robotics/UFS testing system. With a validated finite element model, it will be possible to calculate stress and strain distributions in the ACL and ACL grafts, as well as to develop a database of the in-situ forces for patients of different ages, genders, and sizes. These data are important for one to design preventative programs as well as surgical planning and rehabilitation protocols.

Nevertheless, ligament and tendon research will continue to present many challenges, including the prevention and treatment of tendinopathy as well as ligamentous injuries in addition to the reducing osteoarthritis following ACL injury. Solving these problems will require interdisciplinary and multidisciplinary collaborative research that involves biomedical engineers, biologists, clinicians, plus investigators from many other disciplines (i.e. mathematicians, statisticians and immunologists) to be working together to develop better therapeutic strategies. Ultimately, it is our hope to be able to make ligaments and tendons heal more completely with better tissue quality so that patients can return to both normal daily activities as well as sports.

## Competing interests

The authors declare that they have no competing interests.

## Authors' contributions

HJ, MF and SW all participated in drafting, editing, completion of the manuscript.
